# Validation of a novel procedure for quantification of the formation of phosphoramide mustard by individuals treated with cyclophosphamide

**DOI:** 10.1007/s00280-014-2524-7

**Published:** 2014-07-23

**Authors:** Hans von Stedingk, Hanjing Xie, Thomas Hatschek, Theodoros Foukakis, Andreas Rydén, Jonas Bergh, Per Rydberg

**Affiliations:** 1 Department of Oncology-Pathology, Karolinska Institute, Stockholm, Sweden; 2 Department of Oncology, Karolinska University Hospital, Stockholm, Sweden; 3 Department of Materials and Environmental Chemistry, Stockholm University, Stockholm, Sweden

**Keywords:** Cyclophosphamide, Phosphoramide mustard, Personalized cancer medicine, Hemoglobin adducts, Therapeutic drug monitoring, TDM

## Abstract

**Purpose:**

Use of the patient’s body surface area (mg m^−2^) as a basis for dosing does not take individual variation in metabolic capacity and rate of clearance into account. Here, we evaluated a novel approach for individual monitoring of short-lived cytotoxic agents formed from cytostatic drugs such as cyclophosphamide (CP).

**Methods:**

The accumulated blood dose of the cytotoxic active agent phosphoramide mustard (PAM) formed from CP was measured as a reaction product with hemoglobin (Hb adduct). This adduct, *N*-[2-(2-oxazolidonyl)ethyl]-valyl Hb (OzVal-Hb), was detached from Hb with the adduct FI*R*E procedure™, and the formed analyte was quantified using LC-MS/MS. This dose biomarker for PAM and the analytical procedure was evaluated in accordance with the guidelines on bioanalytical method validation formulated by the European Medicine Agency. The evaluated method was applied to quantify blood dose levels of PAM in female breast cancer patients (*n* = 12) before and after three cycles of polychemotherapy regimes containing CP.

**Results:**

OzVal-Hb, a specific and stable biomarker, could be measured with great sensitivity (lower limit of quantification = 33 pmol g^−1^ Hb), high accuracy (within ±20 %) and good repeatability (CV < 20 %). The inter-individual variability in the blood level of this adduct in women with breast cancer (*n* = 12) who received three doses of CP in combination with one or two other cytostatic drugs was 250 % following the first dose and approximately 150 % after each subsequent dose.

**Conclusions:**

Measurement of the biomarker OzVal-Hb can be used to quantify the short-lived cytotoxic agent PAM in a single blood sample drawn several days after therapy. This procedure may aid in individualizing doses of CP, thereby improving efficacy while both reducing the risk of and increasing the predictability of side-effects.

**Electronic supplementary material:**

The online version of this article (doi:10.1007/s00280-014-2524-7) contains supplementary material, which is available to authorized users.

## Introduction

In general, dosing of cytostatic drugs is based on the patient’s body surface area (BSA, mg m^−2^) or weight (mg kg^−1^), although little evidence actually validates these approaches [1, 2]. The major concern is that such average optimal BSA dosing for a large group of patients does not take into account individual variations in metabolism and rate of clearance.

The cytostatic prodrug cyclophosphamide (CP) must be metabolized to obtain the active agent. This involves hydroxylation in the liver to form the unstable precursor 4-hydroxy cyclophosphamide (4-OH-CP), the rate of which can vary tenfold between individuals [3, 4]. Part of this product diffuses through membranes into the nucleus, where it decomposes to the cytotoxic phosphoramide mustard (PAM) [5]. No clinical assay for direct measurement of PAM is presently available. Instead, the precursor, 4-OH-CP, is commonly measured after extraction and derivatization [3, 4]. Also, acrolein, which is generated when aldophosphamide, the ring-opened tautomer of 4-OH-CP, decomposes to PAM [5–8], has been used to estimate the concentration of PAM. This lack of a clinical assay for determination of the area under the plasma concentration–time curve (AUC) of PAM is problematic, since the difference between the concentration of a cytostatic drug required for efficacy and that which causes severe toxicity to vital organs is often small [8]. Thus, many patients may be under- or overdosed with CP.

A more effective and safer approach is therapeutic dose monitoring (TDM), which involves determination of the internal level of the cytostatic agent in an individual and adjustment of the dose on the basis of this value. However, TDM is rarely employed, since the traditional analytical procedures currently available require multiple blood samples to obtain the AUC. Moreover, metabolites such as PAM, which exhibits a short half-life in patients (14 min [7]), occurs at low concentrations and is difficult to isolate from blood (in this case because of its ionic nature), are exceedingly difficult to quantify.

While the desired cytotoxic effect of PAM arises from its reaction with DNA, this active electrophilic metabolite also reacts with other biomacromolecules, including proteins. Many electrophiles bind covalently to the nucleophilic N-terminal valine residue in hemoglobin (Hb) to form so-called adducts. Since 1986, quantification of N-terminal Hb adducts employing the *N*-alkyl Edman procedure and slightly modified variants thereof has been applied to monitor industrial exposure in humans, as well as low-level chemical exposure in general human populations [9, 10].

**Fig. 1:**
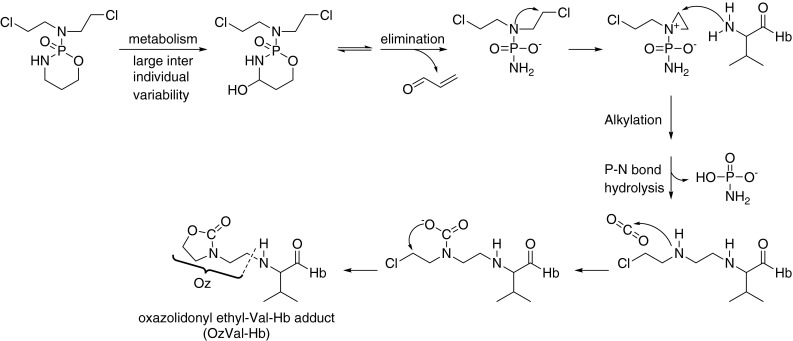
Proposed metabolism of CP to 4-OH-CP, followed by degradation to PAM, which then reacts with the N-terminus of hemoglobin (Hb) followed by incorporation of carbon dioxide to form the stable OzVal-Hb adduct

To date, this approach has not been utilized by oncologists to measure internal doses of chemically reactive drugs/metabolites in humans. One reason for this may be the low rate of sample throughput due to the many time-consuming steps involved, in combination with the complex analytical setup. Thulin et al. [11] developed the *N*-alkyl procedure further to successfully measure protein adducts formed from both CP and nornitrogen mustard (NNM) in mice, as well as from NNM in a hemolysate in vitro. The *N*-[2-(2-oxazolidonyl)ethyl]-valyl Hb (OzVal-Hb) adduct can only be formed from bisalkylating agents, i.e., PAM or its hydrolysis product NNM, as suggested by the mechanism in Fig. 1. Formation of this adduct is favored by a high concentration of CO_2_/HCO_3_ in the blood.

In attempt to overcome the shortcomings of the *N*-alkyl Edman procedure, the adduct FI*R*E procedure™ was developed [12, 13]. Since this procedure was designed and optimized for detection by LC–MS/MS, polar, ionic and thermolabile adducts can be measured directly without prior derivatization [12, 13]. However, its major advantage is the 20-fold higher rate of sample throughput in the complete analytical chain (96 samples processed/3 days), allowing its use in research [14, 15], for screening chemical exposure in more than thousands of newborn infants [16, 17] and, in the very near future, for clinical applications within oncology.

**Fig. 2:**
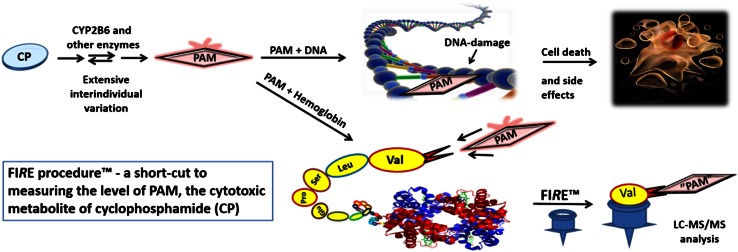
Biotransformation of CP to PAM, which cross-links DNA to achieve the cytotoxic effect and, in a parallel reaction, forms a stable Hb adduct which can be quantified by the FI*R*E procedure™ to obtain a measure of the internal dose of PAM in RBC accumulated over time

Our present aim was to adapt, optimize and validate measurement of OzVal-Hb adducts by the FI*R*E procedure™. The procedure was evaluated for selectivity, carryover, lower limit of quantification (LLOQ), accuracy, precision, matrix effects and stability, in accordance with the guidelines on bioanalytical method validation [18] formulated by the European Medicine Agency. To further evaluate the potential applicability of this method, 12 breast cancer patients were monitored before and after three cycles of dosing with CP. The obtained levels of OzVal-Hb adduct measured after the first dose was compared to hematological markers for toxicity such as the relative decrease of WBC and neutrophils measured day 8 after treatments and Hb levels after dose cycle three versus baseline values. This novel approach to quantifying active cytotoxic compounds such as PAM, that are designed to damage DNA by measuring a stable and specific hemoglobin adduct, is illustrated in Fig. 2.

## Patients and experimental design

### Patients

Twelve patients with breast cancer receiving chemotherapy in connection with a randomized phase III study (PANTHER, EudraCT number 2007-002061-12 and GovTrials number NCT00798070 including over 2,000 patients) were divided equally into two study groups. All patients gave written informed consent prior to enrollment. This study was pre-approved by the Regional Ethics Review Board in Stockholm, Sweden (amendment to SBG2004-1, Dnr: 2009/1023-32).

The patients in group A received tailored therapy; dose adjustment based on the individual evaluation of laboratorial and clinical toxicity, using common toxicity criteria (CTC) at each course. The starting doses were epirubicin 90 mg m^−2^ and cyclophosphamide 600 mg m^−2^ BSA. In connection with the second and third treatments, administered 2 and 4 weeks later, the dose of CP was adjusted upward, to a maximum of 900 mg (*n* = 5) and 1,200 mg m^−2^ (*n* = 4), respectively, and the maximal epirubicin dose was 120 mg m^−2^ BSA. The patients in group B received three BSA-adjusted doses of 5-fluorouracil, epirubicin and cyclophosphamide (F_500_E_100_C_500_) at 3-week intervals followed by docetaxel. Demographic and clinical characteristics of studied patients are given as supplementary data (Supplementary Table 1).

### Blood samples

Blood samples collected from each patient before the first treatment and 14 or 21 days after the first, second and third cycles of dosing were stored at −20 °C until analysis. Commercially available human blood (Karolinska University Hospital, Stockholm, Sweden), stored in the same manner, was used as a control, as well as to obtain a reference. To produce the reference, lysed whole blood was alkylated with 4-OOH-CP (150 µg ml^−1^, 16 h, 37 °C), a commonly used precursor to 4-hydroxycyclophosphamide (4-OH-CP). Subsequent dilution with whole control blood gave samples containing low, medium and high levels of the OzVal-Hb adduct (42, 87 and 162 pmol g^−1^ Hb, respectively).

### Chemicals and reagents

Fluoresceine-5-isothiocyanate (FITC; isomer I. >90 % purity) and the analytical standards fluorescein-5-[4-isopropyl-3-*N*-(2-(2-oxazolidonyl)ethyl)-2-thioxo-imidazolidin-5-one] (OzVal-FTH, 97 % purity), fluorescein-5-[4-^13^C_5_-isopropyl-3-^15^ *N*-(2-(2-oxazolidonyl)ethyl)-2-thioxo-imidazolidin-5-one] (Oz-^13^C_5_^15^N-Val-FTH, 97 % purity) as well as the peptide adducts *N*-[2-(2-oxazolidonyl)ethyl]-valyl-leucyl-anilide (Oz-VLA, 97 % purity) and *N*-[2-(2-oxazolidonyl)ethyl]-^13^C_5_^15^N-valyl-leucyl-anilide (Oz-^13^C_5_^15^N-VLA, 97 % purity) were obtained from Adduct Analys AB (Enebyberg, Sweden). 4-Hydroperoxycyclophosphamide (4-OOH-CP) was a gift from Baxter (Baxter Deutschland GmbH). All other chemicals and solvents employed were of analytical grade.

### Quantification of OzVal-Hb levels utilizing the FI*R*E procedure™

The FI*R*E procedure™ was optimized for quantification of OzVal-Hb adduct as described below. Since this procedure utilizes fluorescein isothiocyanate (FITC) as the Edman reagent, derivatization could be performed directly with unprocessed blood and the analytes formed isolated by protein precipitation and purified on mix-mode SPE columns. The key steps in the measurement of in vivo levels of PAM with the FI*R*E procedure™ are depicted in Supplementary Fig. 1.

For determination of the OzVal-Hb adducts, the Hb concentration in each sample was first measured with an Hb201 + HemoQue instrument (HemoQue, Ängelholm, Sweden) and thereafter, Oz-^13^C_5_^15^N-VLA (20 pmol) was added to the blood (250 µl) as an internal standard. Next, FITC (3 mg in 30 µl dimethylformamide) was added and the samples heated and mixed (40 °C, 750 rpm, 8 h). Acetonitrile (1.5 ml) was subsequently added to precipitate the proteins, followed by centrifugation (5 min, 20,000×*g*; Heraeus Pico 21 Microcentrifuge, Thermo Scientific, Langenselbold, Germany).

Ammonium hydroxide (1 M, 15 µl) was added to the supernatant thus obtained before pouring it into a solid-phase extraction mixed-mode anion-exchange cartridge (Oasis MAX, 60 mg sorbent weight, Waters, Massachusetts, USA). The resin was washed with acetonitrile (1.5 ml), water (1.5 ml) and 0.25 % cyanoacetic acid in acetonitrile/water [3:7 (v/v), 1.5 ml] prior to elution of the analytes with 0.25 % cyanoacetic acid in water/acetonitrile [4:6 (v/v), 1.4 ml]. A gentle stream of air was used to remove all solvents from this eluate and the solid residue then dissolved in water/acetonitrile [7:3 (v/v), 100 µl] prior to analysis (20 µl) with LC/MS.

The LC/MS setup consisted of a Shimadzu Prominence LC 20 system interfaced with a Sciex API 3200 Q-trap instrument. The mobile phase consisting of 0.1 % formic acid in acetonitrile/water [1:1 (v/v)] was passed with isocratic flow (0.12 ml min^−1^) through a X-select HSS T3 column (50 × 4.6 mm, 3 µm) (Waters) or a discovery HS C18 column (150 × 2.1 mm, 3 µm; Supelco Analytical). The analysis was performed in the positive ion mode (ESI+) using multiple reaction monitoring (MRM) at the following transitions: OzVal-FTH *m*/*z* 602.2 → 563.2; 602.2 → 460.2; and Oz-^13^C_5_^15^N-Val-FTH *m*/*z* 608.2 → 563.2; 608.2 → 463.2.

### Optimization of adduct analysis

On the basis of earlier optimization of the FI*R*E procedure™ for the determination of other types of Hb adducts [14, 17], a number of parameters were chosen for examination here. Since ionization of FITC requires one equivalent of base, different amounts of KHCO_3_ (0.050–4.4 mmol g^−1^ Hb) were added to alkylated reference blood samples (*n* = 7, analysis in triplicate) immediately prior to the addition of FITC. Subsequent work-up and analysis were performed as described above.

To evaluate the relationship between the amount of FITC added and yield of OzVal-Hb adduct, alkylated reference blood (250 µl) was incubated (in triplicate) with different amounts of this reagent (0.11, 0.15, 0.20, 0.26, 0.35 and 0.47 mmol g^−1^ Hb) followed by the standard procedure described above.

The yield obtained with different periods of incubation (1, 2, 4, 8, 16 and 24 h, in quadruplicate) at 40 °C followed by storage at −20 °C, and further standard processing (within 24 h of derivatization) was also examined.

For elution of the analytes, cyanoacetic acid, formic acid and trifluoroacetic acid, as well as different relative amounts of the organic phase were compared.

### Validation of our procedure

In accordance with the guidelines on bioanalytical method validation (2011) formulated by the European Medicine Agency [18], our procedure was evaluated for selectivity, carryover, LLOQ, accuracy, precision, matrix effects and stability.

Selectivity was addressed by measuring OzVal-Hb adduct in blood samples from patients (*n* = 12) prior to their treatment with CP, as well as following incubation of control blood with the precursor of PAM, 4-OOH-CP in vitro. For the latter incubations, 250 µl each of six individual blood samples, taken before treatment and not lysed, were incubated (in duplicate) in 2-ml Eppendorf vials with four different concentrations of 4-OOH-CP (1.5, 4.4, 13 and 40 µg ml^−1^; obtained by evaporation of an appropriate amount of a stock solution of 1 mg ml^−1^ in dry THF). These samples were stirred and incubated at 37 °C for 2 h, with parallel incubations of lysed samples, and then stored at −20 °C until analysis.

Carryover was examined by analyzing a blank sample immediately after determination of the highest concentration on the calibration curve, as well as after running the patient samples containing the highest levels of adduct.

Solutions for calibration were prepared by adding the Oz adduct of dipeptides (Oz-VLA; 4, 8, 20, 40, 80, 200 and 400 pmol ml^−1^) and the stably labeled internal standard Oz-^13^C_5_^15^N-VLA (40 pmol ml^−1^ blood) to control blood. The areas under the peaks (OzVal-FTH/Oz^13^C_5_^15^N-Val-FTH ratios) were plotted against concentration of Oz-VLA and the resulting curves drawn by linear regression analysis. The criterion for the LLOQ was a signal-to-noise ratio >10 and a deviation from the nominal value of less than 20 %.

Employing control blood spiked with Oz-VLA at concentrations of 4, 8, 40 and 200 pmol ml^−1^, the within-run accuracy was evaluated by analyzing five samples at each concentration and the between-run accuracy by comparing samples processed on three different days. Precision was determined utilizing these same samples, as well as by repeated analysis of control blood incubated with 4-OOH-CP.

Potential matrix effects were addressed by adding OzVal-FTH (20 and 200 pmol ml^−1^) to processed blood samples (from six individual patients, prior to treatment), after the cleanup procedure and prior to LC–MS analysis, and comparison with standard solutions containing the same concentrations of OzVal-FTH.

The stability of the reference standards was examined by analyzing their retention times and the potential formation of new peaks with LC–UV (254 nm) and LC–MS (ESI+, full scan *m*/*z* 50–1,000).

To determine the influence of storage conditions on the stability of the OzVal-Hb adduct, blood from six patients was first stored at room temperature for up to 7 days (one sample from each patient per day, 1–7 days), then frozen to −20 °C and thereafter subjected to three cycles of freeze–thawing (*n* = 6) or stored for 12 months at −20 °C (*n* = 6) prior to analysis. The potential effect of different levels of Hb (obtained by adding separated red blood cells to plasma from the same individual) in the blood during derivatization and processing was also evaluated (*n* = 6).

### Statistical analysis

Spearman’s correlation coefficients were used to assess correlations between obtained concentrations of OzVal-Hb adduct and hematological markers for toxicity such as relative decrease in white blood cells count and neutrophils measured day 8 and decrease in Hb levels during treatment. Two-sided *p* < 0.05 was considered statistically significant. Data were analyzed using IBM SPSS statistics, version 22 (IBM Corp, New York, USA).

## Results

### Identification of a stable OzVal-Hb adduct formed from PAM

**Fig. 3:**
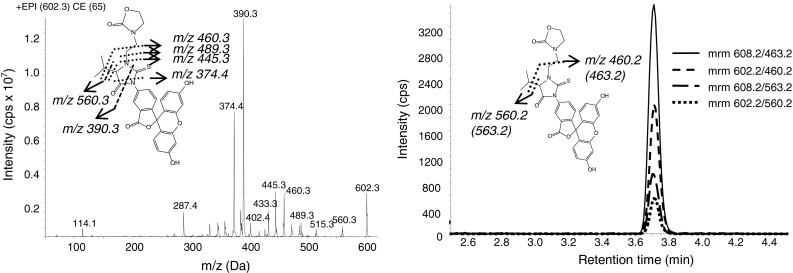
LC-MS/MS (ESI^+^, enhanced product ion scan) spectra of OzVal-FTH illustrating interpretation of the major fragments (*left*). Chromatogram illustrating the MRM transitions for OzVal-FTH and the internal standard Oz-^13^C_5_^15^N-Val-FTH (*right*)

In blood samples from patients treated with CP, as well as in control blood samples incubated with 4-OOH-CP, the OzVal-Hb adduct was easily detected. Following derivatization and detachment with the FI*R*E procedure™, the OzVal-Hb adduct was characterized by LC–MS in the MRM mode and its structure confirmed by comparison with authentic reference standards (Fig. 3). With this approach, the adduct could be detected and quantified in the blood of all the patients after, but not before, the treatment with CP.

### Optimization of the analytical procedure

Altering the buffer capacity of the blood by addition of KHCO_3_ prior to the coupling reaction did not enhance the yield of adduct. Indeed, addition of KHCO_3_ in molar amounts equal to or in excess of FITC (see “Quantification of OzVal-Hb levels utilizing the FI*R*E procedure™” section) actually reduced this yield by enhancing ion suppression during the LC/MS analysis.

Use of 0.26 mmol FITC per g Hb (approximately 3 mg per 250 µl blood) gave the optimal yield, with a 25 % reduction in the level of FITC lowering the yield of OzVal-FTH by 10 % (SD 7 %; Supplementary Fig. 2).

The yield of OzVal-FTH was maximal following 8 h of incubation at 40 °C (Supplementary Fig. 3). This figure also reveals that the ratio of products to internal standard was remarkably similar at all time-points.

Addition of known amounts of OzVal-FTH (5 pmol/250 µl; *n* = 6), before and after processing of blank control samples, revealed that the overall recovery during the cleanup procedure (including protein precipitation, SPE cleaning and evaporation of the solvent in the eluent from the SPE columns) was 74 % (4 % SD) and that ion suppression normalized to the internal standard was insignificant [−3 % (8 % SD)]. This recovery was not improved by eluting the sample from the SPE column with a solution containing more than 60 % acetonitrile or by modification of the pH (with 1 % CNHOAC, 0.25 % formic acid or 0.25 % trifluoroacetic acid) during the cleanup procedure. However, addition of 0.25 % CNHOAC to the solution used for elution reduced the risk of precipitation in the processed samples during storage.

### Validation of the analytical procedure

#### Selectivity

Analysis of solvent blanks and blood samples taken from patients prior to treatment with CP revealed no peaks with retention times similar to those expected for OzVal-FTH or Oz-^13^C_5_^15^N-Val-FTH. Nor was any carryover effect observed when blank samples were injected following analysis of samples containing high levels of the adduct.

**Fig. 4:**
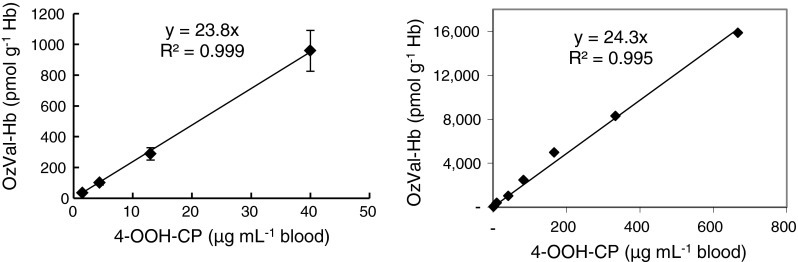
Correlation between formation of the OzVal-Hb adduct in blood samples from six individual patients taken prior to treatment and concentrations of 4-OOH-CP. *Left* levels relevant for CP therapy. *Right* levels from below to above therapeutic doses

Formation of the oxazolidonyl adduct involves addition of carbon dioxide (Fig. 1). This adduct could in theory also be produced when dissolved HCO_3_^−^ reacts as a nucleophile on a formed aziridine intermediate followed by subsequent ring-closure and elimination of water. To clarify whether this step, addition of CO_2_, influences the rate of adduct formation (yield), control blood was incubated with low to very high concentrations of 4-OOH-CP (far above those of clinical relevance) and formation of OzVal-Hb adduct was observed to be linearly dependent on the amount of 4-OOH-CP present, with no decline at the higher concentrations (Fig. 4). Moreover, incubation of clinically relevant concentrations of 4-OOH-CP with six individual samples of fresh blood revealed a virtually identical relationship, with small individual variations (Fig. 4). However, 4-OOH-CP was not stable in freshly distilled dry THF, as indicated by the lower absolute levels of adducts in both patient and control samples analyzed with a solution of 4-OOH-CP that had been stored (approximately 46 % reduction following 30 days of storage at −20 °C). However, the ratio of the adduct levels in patient samples and frozen control blood remained the same (mean ratio 0.52, RSD 12.8 %) even after 30 days storage.

#### The calibration curve

To quantify as accurately as possible, dipeptide adducts (Oz-VLA) were employed for calibration and Oz-^13^C_5_^15^N-VLA as the internal standard and a linear fit applied to obtain the calibration curve. The lowest concentration on this curve was defined as the LLOQ and found to be 4.0 pmol ml^−1^ blood (33 pmol g^−1^ Hb). The LOD (with a signal-to-noise ratio of three) calculated from the signal-to-noise ratio of the LLOQ was approximately 4 pmol g^−1^ Hb. Back calculations of concentration were all within ±15 % of the nominal value.

#### Precision and accuracy

As documented in Table 1, precision with control blood samples spiked with known amounts of Oz-VLA was better than 15 % of the CV in all cases, except for the within-run precision at a concentration equal to the LLOQ, which was 18 %. The between-run and within-run precisions for all control blood samples incubated with 4-OOH-CP were better than 15 %. Employing the Oz-VLA peptide as a surrogate for N-terminal OzVal-Hb, accuracy was found to be within ±15 % of the nominal values, except for the within-run accuracy at LLOQ which was ±16 % of the nominal value.

**Table 1:** Matrix factors (MF) for the OzVal-FTH analytes, precision and accuracy of our analyses of different concentrations of Oz-VLA dipeptide, and precision with control samples incubated with 4-OOH-CP

Oz-VLA peptide [pmol ml^−1^ blood] (relative to the LLOQ)	[4] 1	[8] 2	[40] 10	[200] 50
Between-run precision, CV (%, *n* = 3)	7.5	3.9	8.9	12.6
Within-run precision, CV (%, *n* = 6)	18.0	11.1	13.6	9.2
Between-run accuracy, % (CV, *n* = 3)	105 (7.8)	110 (4.2)	104 (9.3)	97.1 (12.2)
Within-run accuracy, % (CV, *n* = 6)	116 (19.1)	91.6 (9.2)	95.2 (11.8)	95.5 (8.0)

Blood incubated with 4-OHCP adduct level	Low	Medium	High	
Between-run precision, CV (%, *n* = 3)	12.9	7.0	6.0	
Within-run precision, CV (%, *n* = 4)	2.6	6.5	3.5	

Matrix factor at	[20 pmol ml^−1^]	CV (%, *n* = 6)	[200 pmol ml^−1^]	CV (%, *n* = 6)
OzVal-FTH	0.66	14.9	0.61	3.4
Oz-^13^C_5_^15^N-Val-FTH	0.67	17.7	0.59	7.5
Normalized to the internal standard	0.99	12.6	1.06	9.0

#### Matrix effects

As evaluated with processed control blood samples from six individual patients containing low and high concentrations of the analyte, a matrix effect for both the analyte and internal standard could not be avoided, despite cleanup with the analytical column prior to MS. However, since this matrix effect normalized to the internal standard was close to one, with an acceptable CV (<±15 %; Table 1), this factor is not expected to influence quantification of the absolute levels to any significant extent.

#### Stability and robustness

When blood samples (250 µl, *n* = 6) from patients treated with CP were left at room temperature for as long as 7 days before freezing, no significant effect (<20 %) on the adduct levels detected was observed. Moreover, the OzVal-Hb adduct was stable during three cycles of freeze/thawing (<20 % change; *n* = 6). Following storage for 12 months at −20 °C (*n* = 6), the ratio between the level of analyte and internal standard was only altered by 3.1 % (CV = 19 %).

Samples must be lysed to allow FITC to react with the N-terminus of Hb, and the relative effectiveness of freeze/thawing with or without subsequent sonication in this connection were compared. Sonication for 5, 15, 30 or 60 min after lysing and before further processing had no influence on the results obtained (Pearson’s correlation = −0.14, *p* = 0.56, *n* = 6).

Once OzVal-FTH was formed by derivatization, neither storage for 24 h at −20, +6 or +37 °C, nor precipitation with acetonitrile at room temperature prior to further cleanup influenced the level of adduct detected (*t* tests, *p* ≥ 0.43).

Dilution of red blood cells from patients with their own plasma to obtain different concentrations of Hb had no apparent effect on the level of OzVal-Hb adduct subsequently detected (Supplementary Fig. 4).

Reanalysis of processed samples following storage for 72 h at −20 °C or in an autosampler at room temperature altered the results obtained by <20 %.

Analysis by LC–UV–MS revealed no degradation products of the reference standard Oz-VLA or Oz-^13^C_5_^15^N-VLA following storage in 2-propanol/H_2_O (3:7) at −20 °C or +8 °C for 6 months or at −20 °C for 12 months.

#### Levels of the OzVal-Hb adduct in patients being treated with CP

**Fig. 5:**
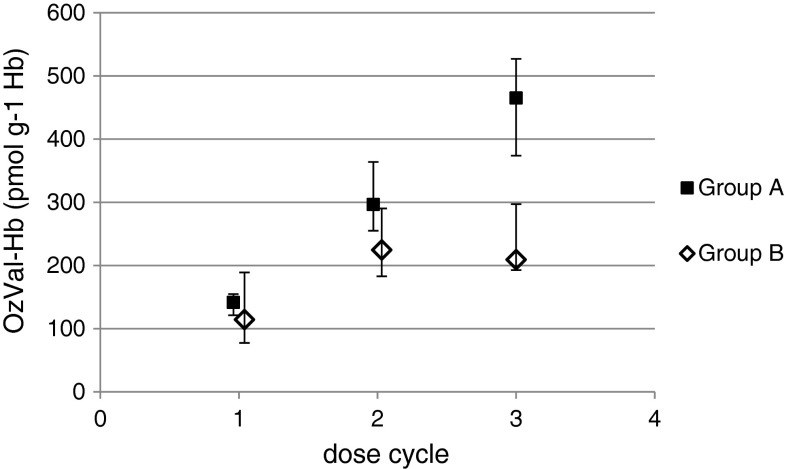
Levels of OzVal-Hb adducts in group A and B following three cycles of CP dosing, the values presented are means ± min–max (the *error bars*) for six individuals per group

The levels of OzVal-Hb adducts in blood collected from patients before treatment and after three subsequent cycles of dosing with either 500 mg CP m^−2^ BSA (group B, *n* = 6) or one 600 mg dose of CP m^−2^ BSA followed by biweekly stepwise increases based on individual toxic response (group A, *n* = 6) were determined. Following the first dose, the mean level of adducts differed by 24 % between groups A and B, in agreement with the amount of CP administered. The difference between the patients with the highest and lowest values was approximately 250 % after the first dose and 150 % after each subsequent dosing cycle (Fig. 5). The patients exhibiting lower or higher levels after the first dose continued to do so following the second and third dose cycles as well. No background levels or interfering peaks were present in the samples collected prior to treatment with CP.

#### Relation between measured OzVal-Hb adduct level and hematologic variables

In dose group B, a negative trend was observed between OzVal-Hb adduct measured after the first dose and relative decrease of WBC (Rs = −0.66, *p* = 0.16, *n* = 6) and neutrophils (Rs = −0.46, *p* = 0.35, *n* = 6) measured day 8 after treatments and Hb levels after dose cycle three versus baseline values (Rs = −0.83, *p* = 0.04, *n* = 6). In group A, there was no clear trend observed. Data are given in Supplementary Table 2.

## Discussion

We have demonstrated that the FI*R*E procedure™ can be applied to quantify the OzVal-Hb adduct as an indicator of the level of the reactive and cytotoxic metabolite PAM formed in vivo from CP. Our custom designed procedure is robust in terms of selectivity, linearity, accuracy, precision and stability and thus fulfills all the criteria for validation presented in the guidelines on bioanalytical method validation (2011) formulated by the European Medicine Agency.

The optimal amount of FITC found here represents a compromise between a good yield of thiohydantoin and occurrence of side reactions that lead to ion suppression. Although a reaction time of 16 h provided the highest yield of analyte, the ratio of the level of analyte to that of the internal standard was independent of reaction time. This indicates that use of the dipeptide containing a stable isotope (Oz-^13^C_5_^15^N-VLA) represents a robust approach to quantification.

To form the OzVal-Hb adduct, PAM first reacts with the N-terminal valine in Hb and then CO_2_ is incorporated, which subsequently deactivates the last reactive functionality through alkylation of the carboxylate, rendering the OzVal-Hb adduct chemically stable under physiological conditions. In all three individual blood samples examined, the formation of OzVal-Hb adduct was linearly proportional to the amount of 4-OOH-CP present. In order to compensate for degradation of 4-OOH-CP dissolved in a stock solution (dry THF, stored at −20 °C <5 days), reference blood samples were incubated in parallel.

Presumably, more than 99 % of the 4-OH-CP formed from 4-OOH-CP had reacted after 2 h of incubation. The patient samples were fresh whole blood, whereas the control samples were lysed blood, which resulted in a ratio of 0.52 between the adduct levels in these samples. No background levels of OzVal-Hb adducts or interfering peaks were observed.

Some specific adaptation of the guidelines for validation was necessary, since a well-defined Oz-Hb standard (containing a known level of adduct) is not commercially accessible. As a surrogate, the peptide Oz-VLA was employed for quantification and determination of accuracy. The studies on precision were also complemented by repeated measurements of alkylated reference samples. With both of these approaches, the results were acceptable according to the guidelines.

In both alkylated reference blood samples and blood samples from patients, the OzVal-Hb adduct is stable under the conditions used here for sampling and processing. Moreover, these adducts proved to be stable even after storage of blood at room temperature for 7 days. This stability will facilitate routine sampling in the clinic.

In all of the patients treated with CP, the blood level of the OzVal-Hb adduct increased after each cycle of dosing in the stepwise manner expected. A stable Hb adduct will disappear with the same kinetics as the erythrocytes, which have a life span of approximately 120 days [10]. Thus, if the time-points for administration of the drug and sampling are known, the relative AUC of the electrophile can be calculated from the levels of Hb adduct (see Supplementary Fig. 5).

The 24 % difference between the adduct level in the patients in groups A and B following the first administration of CP agrees well with the difference in initial dose (600 vs. 500 mg m^−2^ BSA, respectively). The more pronounced differences following dosing cycles two and three reflect the higher dose administered at shorter intervals in the case of group A. The difference in adduct levels between the individuals with the highest and lowest values was 250 % after the first dose and approximately 150 % after each subsequent cycle of dosing. The patients exhibiting lower or higher levels after the first dose continued to do so following the second and third dose cycles as well.

Due to the limited number of patients, the obtained data for correlations between the OzVal-Hb adduct and hematologic variables (given in Supplementary Table 1) should not be over interpreted. However, a negative relationship was observed in group B who received fixed BSA dosing. These patients had also the largest individual variation of the OzVal-Hb adduct levels (77–189 pmol g^−1^ globin) which facilitates the search for correlations between biomarker level and toxicity in relation to patients in group A who were more uniform (121–155 pmol g^−1^ globin).

The large interindividual variability in capacity to metabolize CP to 4-OH-CP has been extensively characterized [3, 19]. Since direct measurement of PAM is complicated, previous investigations have involved in the determination of its precursor, 4-OH-CP, after cleanup and derivatization. Determination of the AUC for 4-OH-CP itself has required repeated sampling during the period of several hours when the drug is active [3, 19].

Quantification of the OzVal-Hb adduct with the FI*R*E procedure™ as described here provides a novel analytical tool for the determination of the accumulated blood dose of PAM in RBC, a valuable “short-cut” to obtaining the internal dose of the active cytotoxic agent from a single blood sample. The small amount of blood needed (0.5 ml) also enables applications toward infants and small children. This is an important area of research, that due to methodical and ethical reasons (by applying conventional approaches), consists of limited data. Furthermore, the fact that the sample has taken a long time (days) after the drug has been excreted, enables measurements at a time that is convenient for both the patient and his/her caregivers. The next step will be to demonstrate the clinical usefulness of this novel approach, and such studies are now ongoing in our laboratory.

There are already numerous indications that this approach can greatly improve individualization of CP dosing. One strategy for overcoming the shortcomings of BSA dosing is exemplified by the PANTHER study in which careful monitoring of the degree of toxic side-effects allowed effective adjustment of the dose in well-defined steps. With the TailorDose™ strategy proposed here, measurement of the accumulated blood dose of PAM in RBC in combination with parameters of toxicity could aid in making more precise decisions concerning subsequent dosing with CP, as well as concerning whether the dose of only one of the drugs in a combination treatment should be altered.

For the individual patient, this means, of course, more effective therapy, greater safety and a reduced risk of severe side-effects such as immunodepletion. Moreover, the methodology described here can be used to identify pharmacogenomic relationships and provide other types of accurate data.

## Conclusion

Here, we describe a novel analytical procedure for quantification of OzVal-Hb adduct formed from PAM, the cytotoxic metabolite of CP. This method was validated and proven to be robust in terms of selectivity, stability, accuracy and precision. Blood levels of OzVal-Hb adduct in two groups of patients, one receiving a constant and the other an increasing dose of CP, were clearly related to the dose administered. The adduct level rose after each dosing cycle in the manner expected for a stable biomarker whose biological half-life follows the kinetics of erythrocytes. This approach offers considerable promise as a novel clinical tool for accurate determination of effective dosing, information that can serve as a basis for individual adjustment of the dose of CP, thereby reducing the risk of severe side-effects and improving the efficacy of treatment. We are now evaluating the clinical value of this new approach in a larger study (150 patients), the results of which will be published in the near future.

## Electronic supplementary material

Below is the link to the electronic supplementary material.

Supplementary material 1 (PDF 425 kb)
