# Supplementary material for: Validation of a novel procedure for quantification of the formation of phosphoramide mustard by individuals treated with cyclophosphamide
Source: Cancer Chemother Pharmacol. 2014 Jul 23;74(3):549–58. doi: 10.1007/s00280-014-2524-7 (PMC4143677; doi:10.1007/s00280-014-2524-7)
Supplement: Supplementary file 1 — Supplementary material 1 (PDF 425 kb) [file 280_2014_2524_MOESM1_ESM.pdf]

Supplementary Figures and Tables

Validation of a novel procedure for quantification of the formation of phosphoramide mustard by individuals treated with cyclophosphamide

Hans von Stedingk[1], Hanjing Xie[1,2], Thomas Hatschek[1,2], Theodoros Foukakis[1,2], Andreas Rydén[3], Jonas Bergh[1,2], Per Rydberg[1]

[1] Department of Oncology-Pathology, Karolinska Institute, Stockholm, Sweden
[2] Department of Oncology, Karolinska University Hospital, Stockholm, Sweden
[3] Department of Materials and Environmental Chemistry, Stockholm University, Stockholm, Sweden

Corresponding author: Per Rydberg, tel: +46(0)707471346, email: per.rydberg@ki.se

Journal: Cancer Chemotherapy and Pharmacology

Supplementary Fig. 1

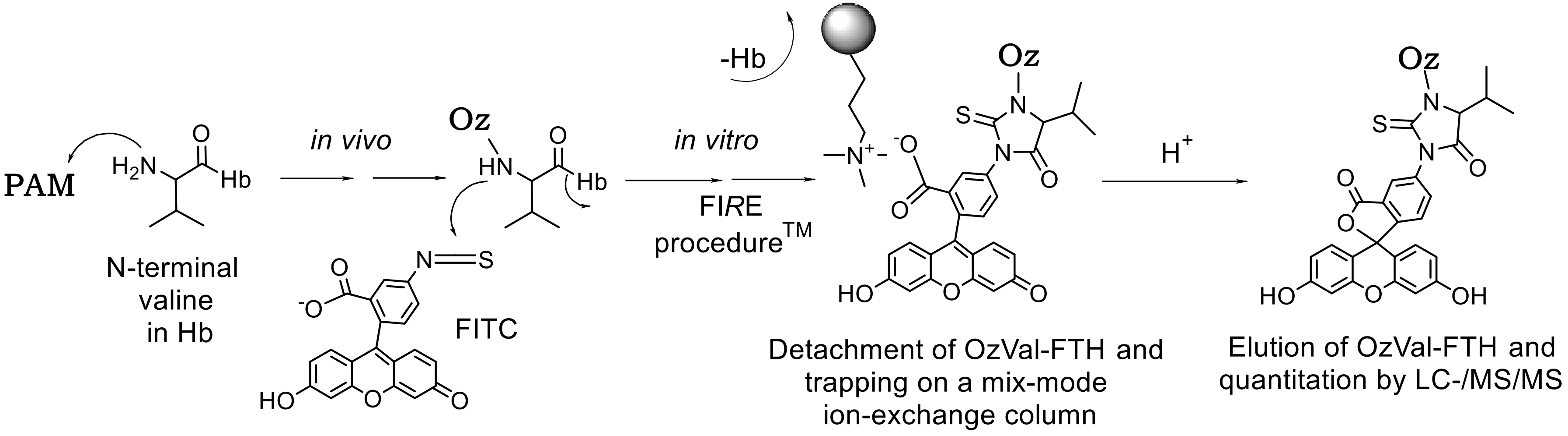

Figure 1: Utilization of the FI*R*E procedure™ to measure OzVal-Hb, an indicator of *in vivo* levels of PAM.

Supplementary Fig. 2

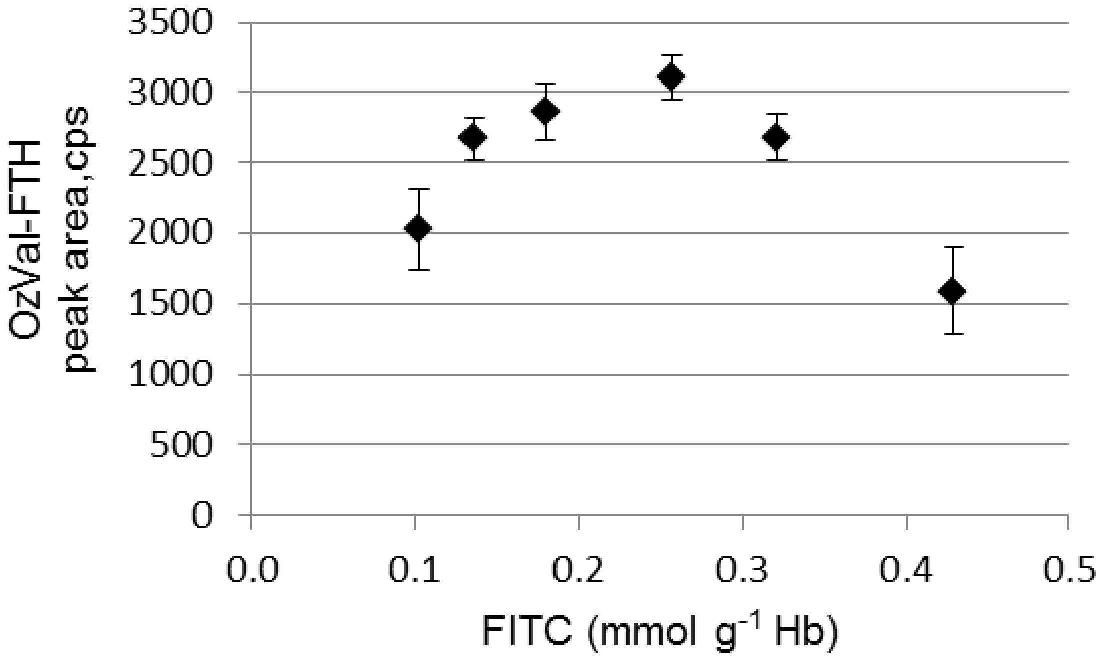

Figure 2: Relationship between the yield of OzVal-FTH and amount of FITC present during derivatization. The values presented are means ± SD (the error bars) for 6 independent samples analyzed in triplicate.

Supplementary Fig. 3

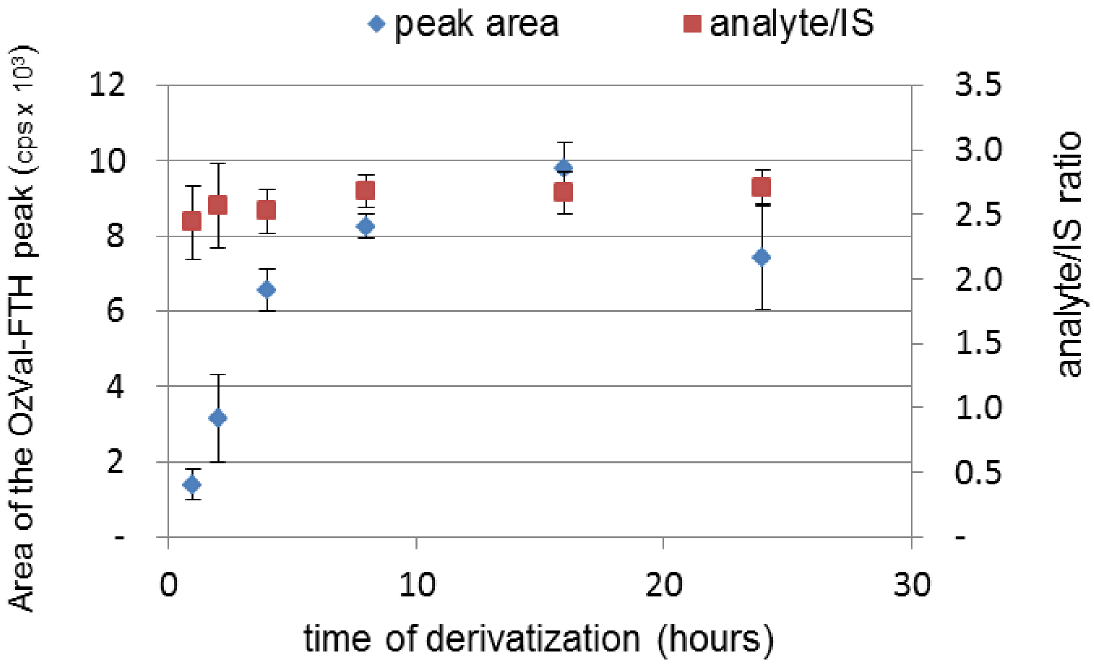

Figure 3: Relationship between the time of derivatization at 40°C and yield of OzVal-FTH (left y-axis) and the ratio of OzVal-FTH produced to the amount of internal standard (IS) added (right y-axis). The values shown are means ± SD (error bars) for 6 independent blood samples analyzed in quadruplicate.

Supplementary Fig. 4

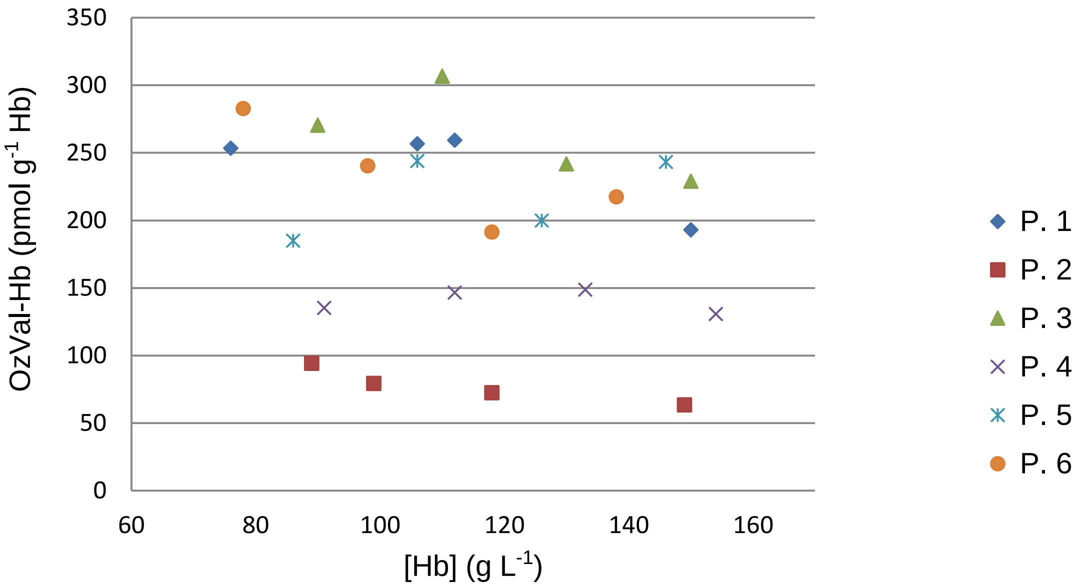

Figure 4: Levels of OzVal-Hb in blood samples from six patients (P1 – 6), diluted to contain different concentrations of Hb.

Supplementary Fig. 5

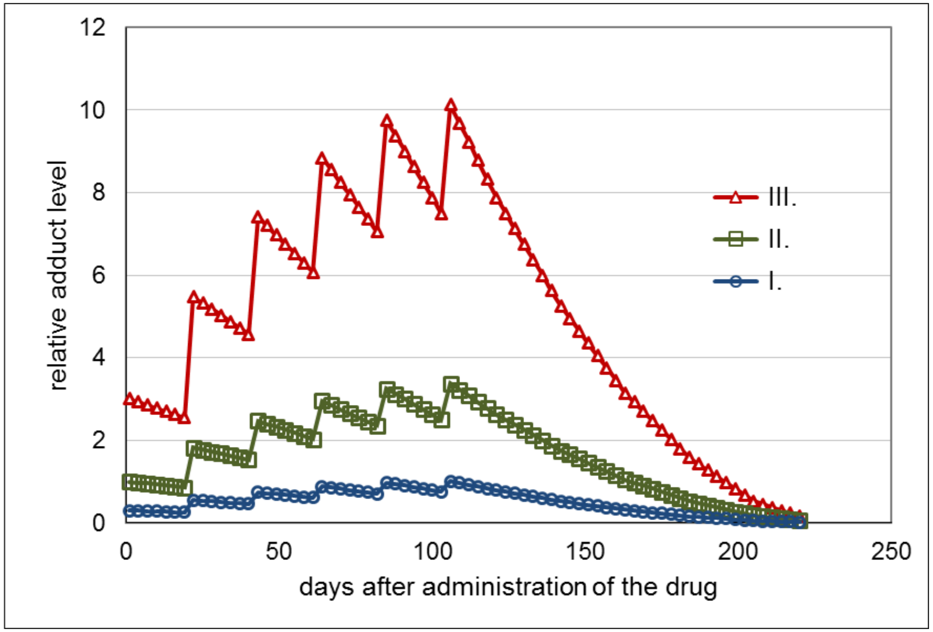

Figure 5: Theoretical AUCs for three individuals producing low (I), medium (II) or high (III) levels of a cytotoxic (and reactive) agent during six cycles of dosing at three week-intervals. The adduct level obtained (e.g., OzVal-Hb) reflects the accumulated blood level minus the rate of elimination, which is determined by the lifespan of the erythrocytes[1].

note[1]: Calculated according to the reference S.M. Osterman-Golkar, H.W. Vesper, J Diabetes Complications, 20, 2006, 285.

Supplementary Table 1.

Table 1: Demographic and selected clinical characteristics of studied patients, mean values presented with min, max values within bracket.

	Group A (*n*=6)	Group B (*n*=6)	All (*n*=12)
Age (years)	54 (48-67)	54 (36-70)	54 (36-70)
Weight (Kg)	72 (56-92)	77 (45-95)	75 (45-95)
BSA (m^2)	1.80 (1.55-2.00)	1.85 (1.44-2.00)	1.87 (1.44-2.00)
OzVal-Hb after cycle 1 (pmol/g Hb)	142 (121-155)	114 (77-189)	128 (77-189)
OzVal-Hb after cycle 2 (pmol/g Hb)	296 (255-364)	225 (183-290)	260 (183-364)
OzVal-Hb after cycle 3 (pmol/g Hb)	465 (374-527)	251 (193-297)	368 (193-527)
Hb baseline (g/L)	130 (119-139)	133 (124-140)	131 (119-140)
Hb after cycle 3 (g/L)	103 (86-112)	114 (99-127)	108 (86-127)
WBC, baseline (10^9/L)	6.2 (4.8-9.1)	6.4 (3.8-9.3)	6.3 (3.8-9.3)
WBC day 8, cycle 1 (10^9/L)	2.6 (0.9-5.2)	2.4 (1.1-6.3)	2.5 (0.9-6.3)
Neutrophiles, baseline (10^9/L)	3.7 (2.8-6.3)	4.1 (2.0-7.1)	3.9 (2.0-7.1)
Neutrophiles day 8, cycle 1 (10^9/L)	1.3 (0.2-3.2)	1.1 (0.1-4.0)	1.2 (0.1-4.0)

Supplementary Table 2.

Table 2: Spearman correlations between OzVal-Hb adduct levels after first dose and hematologic variables

	n	Rs	p
All			
WBC day 8/WBC at baseline	12	-0,29	0,35
neutrophiles day 8/neutrophiles at baseline	12	-0,13	0,68
Hb after 3rd dosecycle / Hb at baseline	12	-0,44	0,15
Group B			
WBC day 8/WBC at baseline	6	-0,66	0,16
neutrophiles day 8/neutrophiles at baseline	6	-0,46	0,35
Hb after 3rd dosecycle / Hb at baseline	6	-0,83	0,04
Group A			
WBC day 8/WBC at baseline	6	-0,26	0,62
neutrophiles day 8/neutrophiles at baseline	6	-0,14	0,79
Hb after 3rd dosecycle / Hb at baseline	6	0,14	0,79
